# De novo pathogenic *DNM1L* variant in a patient diagnosed with atypical hereditary sensory and autonomic neuropathy

**DOI:** 10.1002/mgg3.961

**Published:** 2019-09-01

**Authors:** Maja Tarailo‐Graovac, Farah R. Zahir, Irena Zivkovic, Michelle Moksa, Kathryn Selby, Sunita Sinha, Corey Nislow, Sylvia G. Stockler‐Ipsiroglu, Ruth Sheffer, Ann Saada‐Reisch, Jan M. Friedman, Clara D. M. van Karnebeek, Gabriella A. Horvath

**Affiliations:** ^1^ Centre for Molecular Medicine and Therapeutics BC Children's Research Institute University of British Columbia Vancouver Canada; ^2^ Departments of Biochemistry, Molecular Biology and Medical Genetics Cumming School of Medicine University of Calgary Calgary AB Canada; ^3^ Alberta Children's Hospital Research Institute University of Calgary Calgary AB Canada; ^4^ Department of Medical Genetics University of British Columbia Vancouver Canada; ^5^ University of British Columbia Vancouver Canada; ^6^ Department of Microbiology and Immunology University of British Columbia Vancouver Canada; ^7^ Department of Pediatrics Division of Pediatric Neurology University of British Columbia Vancouver Canada; ^8^ Faculty of Pharmaceutical Sciences University of British Columbia Vancouver Canada; ^9^ Department of Pediatrics Division of Biochemical Diseases University of British Columbia Vancouver Canada; ^10^ Department of Genetics and Metabolic Diseases Hadassah‐Hebrew University Medical Center Jerusalem Israel; ^11^ Department of Pediatrics Centre for Molecular Medicine and Therapeutics BC Children's Research Institute University of British Columbia Vancouver Canada; ^12^ Present address: College of Science and Engineering Hamad Bin Khalifa University Doha Qatar; ^13^ Present address: Departments of Pediatrics and Clinical Genetics Amsterdam University Medical Centers Amsterdam The Netherlands

**Keywords:** *DNM1L*, epileptic encephalopathy, HSAN, intradermal histamine test, self‐injury, whole genome sequencing

## Abstract

**Background:**

Profiling the entire genome at base pair resolution in a single test offers novel insights into disease by means of dissection of genetic contributors to phenotypic features.

**Methods:**

We performed genome sequencing for a patient who presented with atypical hereditary sensory and autonomic neuropathy, severe epileptic encephalopathy, global developmental delay, and growth hormone deficiency.

**Results:**

Assessment of the variants detected by mapped sequencing reads followed by Sanger confirmation revealed that the proband is a compound heterozygote for rare variants within *RETREG1* (*FAM134B*), a gene associated with a recessive form of hereditary sensory and autonomic neuropathy, but not with epileptic encephalopathy or global developmental delay. Further analysis of the data also revealed a heterozygous missense variant in *DNM1L*, a gene previously implicated in an autosomal dominant encephalopathy, epilepsy, and global developmental delay and confirmed by Sanger sequencing to be a de novo variant not present in parental genomes.

**Conclusions:**

Our findings emphasize the importance of genome‐wide sequencing in patients with a well‐characterized genetic disease with atypical presentation. This approach reduces the potential for misdiagnoses.

## 1 INTRODUCTION

High throughput sequencing offers an opportunity to analyze the genome of an individual (whole genome sequencing [WGS]) or a protein‐coding subset of a genome in a single test (whole exome sequencing [WES]). The provision of genome‐wide data from a single screen has played an important role in diagnosing rare disease patients with complex phenotypes (Posey et al., 2017; Tarailo‐Graovac et al., 2016). An emerging challenge is interpreting the genetic results and clarifying if the atypical phenotypes are an expanded clinical presentation of an already suspected monogenic disease, an unsuspected monogenic disease, or do they result from two or more distinct genetic conditions with overlapping (blended) or discrete (composite) clinical features? Population databases of untargeted populations (e.g. gnomAD) play an important role in interpretation of genomic findings (Lek et al., 2016) where thorough review of the findings is essential (Tarailo‐Graovac, Zhu, Matthews, Karnebeek, & Wasserman, 2017).

Hereditary sensory and autonomic neuropathy (HSAN) are a heterogeneous group of disorders characterized by progressive degeneration of the peripheral nervous system and presenting with prominent sensory and autonomic symptoms. Here, we report a proband presenting with a phenotype resembling an atypical form of HSAN. In addition to severe sensory and autonomic neuropathy, the patient also presented with progressive neurodegeneration, profound global developmental delay, and epileptic encephalopathy, CNS features that are previously not reported in HSAN patients. Using a singleton‐WGS analysis, we identified rare biallelic variants in *RETREG1* (also known as *FAM134B* [MIM 613114]) and later, a rare de novo heterozygous variant in *DNM1L* (MIM 603850). After careful review of the variants and other patients with *DNM1L* deficiency, we conclude that the pathogenic de novo variant is the sole cause of the phenotypic features in our patient (including HSAN‐like phenotype), rather than a composite effect of two rare disorders.

## 2 METHODS

### 2.1 Subjects

The patient and the family were initially enrolled in to the Department of Medical Genetics GARD study for rare disease gene discovery (UBC IRB approval H09‐01228) and then subsequently enrolled into TIDEX gene discovery study (UBC IRB approval H12‐00067). They provided informed and written consent for sample collection, WGS, data analysis, and publication of the current case report. Detailed clinical presentation of the proband is available in the Data S1. In brief, the patient presented with developmental delay, decreased pain sensitivity, self‐mutilation, decreased tear production, and autonomic instability. He later developed dystonia, spasticity, and severe epileptic encephalopathy. He had an abnormal intradermal histamine test. About 0.5 ml of histamine phosphate 1:1,000 was injected intradermally in the flexor surface of the forearm, and the reaction read in 5 min. A normal response to injection is the appearance of a wheal usually 1 cm in diameter, with a zone of erythema (flare) around it, approximately 3 cm in diameter. In patients with hereditary sensory neuropathies, the wheal is not surrounded by a flare response.

### 2.2 WGS** and confirmation**

Genomic DNA was isolated using standard protocols and singleton WGS (proband only) was sequenced on an Illumina HiSeq 2500 (University of British Columbia). Our default semiautomated bioinformatics pipeline was used to analyze the data (Tarailo‐Graovac et al., 2016). Confirmation of the variants identified using WGS, as well as segregation with the disease was validated using Sanger sequencing at the CMMT/BCCHRI DNA Sequencing Core Facility (for detailed description of our WGS protocols please see Data S1).

## 3 RESULTS

**Figure 1:**
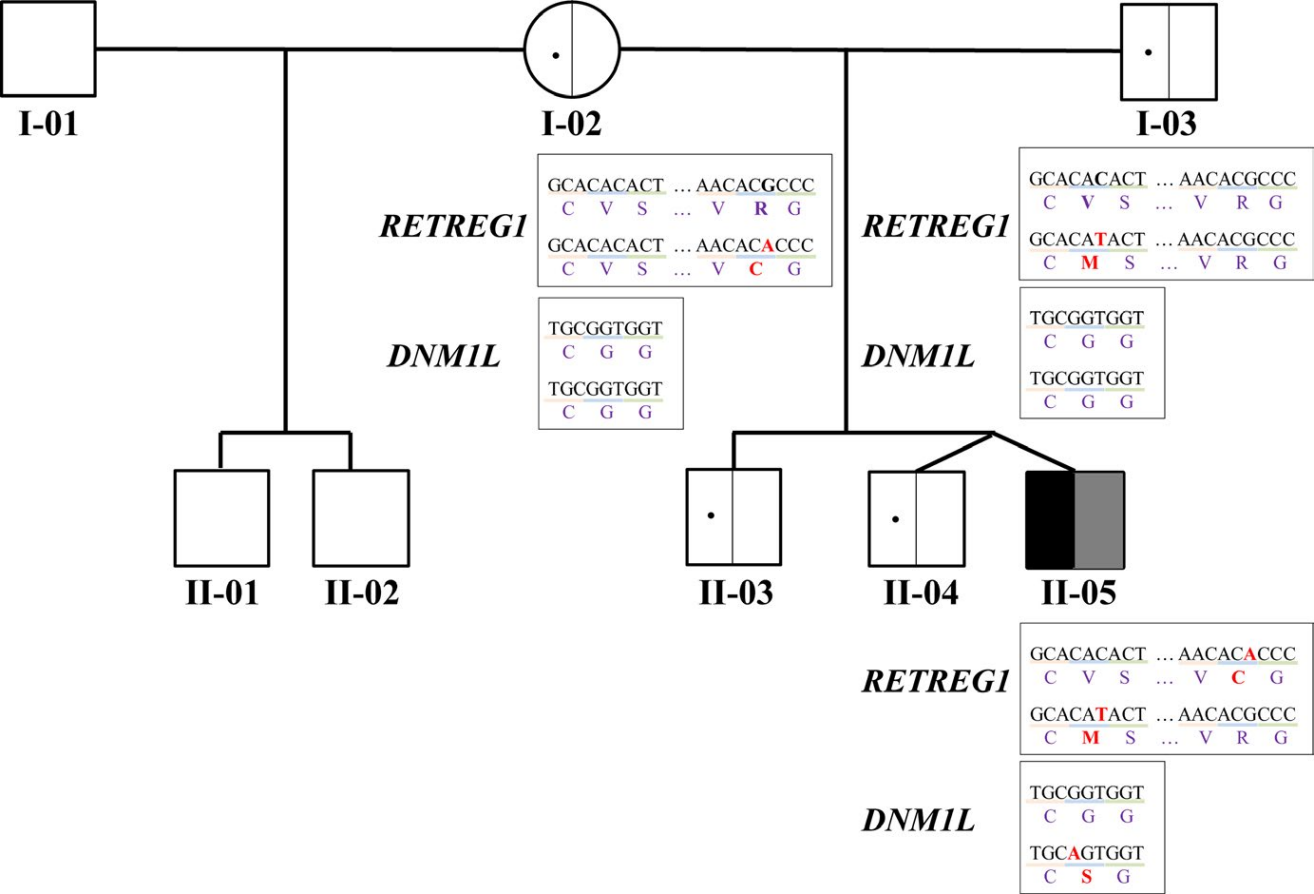
A family with an isolated case of an atypical sensory and autonomic neuropathy combined with severe epileptic encephalopathy and global developmental delay. For the simplicity, the neuropathy phenotype is denoted in black, while the CNS phenotype in gray in the affected proband (II‐05). *RETREG1* variant carrier status in unaffected parents as confirmed by Sanger resequencing is depicted in left half of the symbol. The DNA (reference sequence from Human Genome GRCh37/hg19 assembly) and amino acid changes are depicted as well

The WGS analysis of the proband (Figure 1) revealed rare variants in only one known HSAN‐associated gene, *RETREG1* (HSAN type IIB, an autosomal recessive condition [MIM 613115]) (Kurth et al., 2009). The proband is a compound heterozygote for two missense variants, validated by Sanger resequencing as compound heterozygous (Figure 1), of *RETREG1* (NM_001034850; NP_001030022): (c.607G>A; p.(Val203Met)) and (c.379C>T; p.(Arg127Cys)). Both of the variants are rare according to dbSNP (v.150), NHLBI ESP, our in‐house database and gnomAD (Tarailo‐Graovac, Zhu, et al., 2017). Both of these variants affect conserved amino acids; however, while most of the tools predict the effects of the variants to be deleterious [e.g., high Combined Annotation Dependent Depletion (CADD, v1.3) (Kircher et al., 2014) scores (24.8 for p.(Val203Met); 26.0 for p.(Arg127Cys)), and probably damaging scores by PolyPhen2 (Adzhubei, Jordan, & Sunyaev, 2013)], other tools like SIFT (Kumar, Henikoff, & Ng, 2009) predict the variants to be tolerated. The evidence regarding the clinical significance of these variants, however, suggests that these are likely/benign. One of the variants is classified as likely benign by at least one clinical lab and the other is recorded as benign by all the submitting labs in the ClinVar (Landrum, Lee, & Benson, 2016). The p.(Arg127Cys) variant has been observed in eight homozygous individuals in gnomAD and the p.(Val203Met) variant in one homozygous individual. These observations combined with the fact that, to date only loss of function *RETREG1* variants have been described in HSAN type IIB*,* lead to “likely benign” classification of both variants using the ACMG (American College of Medical Genetics and Genomics) guidelines (Richards, Aziz, & Bale, 2015).

Next, we also identified a heterozygous variant, confirmed de novo variant, in *DNM1L* (Figure 1). A dominant negative mutation in *DNM1L* encoding the dynamin‐like protein 1 was reported in 2007 by Waterham et al. in a patient with a lethal defect of mitochondrial and peroxisomal fission (Waterham et al., 2007). Recent publications further report developmental delay, pain insensitivity, and mitochondrial respiratory chain complex IV deficiency phenotypes (Sheffer et al., 2016); refractory epilepsy (Vanstone et al., 2016); slowly progressive mild neurological impairment (Nasca et al., 2016), and childhood onset epileptic encephalopathy (Fahrner, Liu, Perry, Klein, & Chan, 2016) in patients with *DNM1L* variants.

The heterozygous *DNM1L* (NM_012062; NP_036192) variant (c.1084G>A; p.Gly362Ser) identified here is predicted to be damaging by all the tested tools (Adzhubei et al., 2013; Kircher et al., 2014; Kumar et al., 2009), it affects a conserved amino acid, has not been observed in the NHLBI ESP, our in‐house database, or gnomAD (Tarailo‐Graovac, Zhu, et al., 2017) and according to the ACMG guidelines is classified as pathogenic (Richards et al., 2015). It has been recently reported as a pathogenic variant, identified in a patient with chronic neurological disorder, postnatal microcephaly, developmental delay, decreased respiratory chain complex IV activity, and pain insensitivity (Sheffer et al., 2016). This patient was also suspected initially to have HSAN and at the age of 1 year was examined by a Familial Dysautonomia expert. Intradermal histamine test was pathologically similar to our patient and corneal reflexes were reduced. His sequencing data, however, unlike the genome data of our patient revealed no rare variants in *RETREG1* or other known HSAN‐associated genes (Sheffer personal communication). Furthermore, a heterozygous de novo variant affecting the same amino acid (c.1085G>A; p.(Gly362Asp)) has also been reported in a 7‐year‐old boy with encephalopathy (Vanstone et al., 2016). The conserved p.Gly362 amino acid is located in the domain that is important for homo‐oligomerization and was suggested to result in a dominant negative effect (Vanstone et al., 2016).

## 4 DISCUSSION

The diagnosis of HSAN of unknown type was entertained in this patient from very early on, based on an abnormal intradermal histamine test and the phenotype consistent with painless automutilation. Mutations in *RETREG1* were identified first in 2009 to cause HSAN (Kurth et al., 2009), and spectrum of clinical presentations described since (Data S1), but none of the reported patients presented with CNS features akin to our patient.

Thus, after we identified the missense variants in *RETREG1,* a gene that fits well with the patient's HSAN phenotype, we continued the search for genome changes that could explain the CNS phenotype in our patient. During the prolonged ICU admission of our patient, the diagnosis of mitochondrial disease was entertained but the patient was too unstable for a muscle biopsy under general anesthesia. Identification of a de novo* DNM1L* variant from genome data was considered an excellent fit and was further reinforced with reports on the same (p.Gly362Ser) (Sheffer et al., 2016) or similar (p.Gly362Asp) (Vanstone et al., 2016) de novo* DNM1L* variants in patients presenting with refractory epilepsy published shortly after *DNM1L* discovery in our patient. Recurrent observation of a de novo variation in the Gly362 amino acid in DNM1L deficiency patients suggests an important role of this particular amino acid in the pathophysiology of the disease.

The case presented here is an excellent example of the emerging challenges in clinical genetics with respect to interpretation of complex phenotypes that may be due to either the occurrence of more than one genetic disorder in an individual, or new phenotypic expansion, or complete misdiagnosis in the first place. What initially seemed to be a case of an expanded phenotypic spectrum of RETREG1 deficiency with the identification of the pathogenic *DNM1L* and additional information on the other *DNM1L* patient became in fact an expansion of the DNM1L spectrum. We conclude that the identified rare missense *RETREG1* variants do not contribute to the HSAN phenotype. The pain insensitivity has been observed in one patient with the same *DNM1L* variant (Sheffer et al., 2016), however abnormal intradermal histamine test has never been described, although it was later confirmed by the authors (Sheffer personal communication). However growth hormone deficiency has not been reported before in patients with *DNM1L* variants. Neither of these two patients had evidence of peroxisomal dysfunction. Our findings further illustrate the importance of genome‐wide sequencing, WGS in this case, in accurate diagnosis (Tarailo‐Graovac, Wasserman, & Van Karnebeek, 2017). Had we only applied a gene‐centric approach focused on HSAN‐implicated genes, we might either have interpreted this patient as undiagnosed, consider erroneously that the *RETREG1* variants are disease causing, and suggest an expansion of the phenotypic spectrum associated with biallelic *RETREG1* mutations. On the contrary, we have now expanded the phenotypic spectrum of conditions associated with this *DNM1L* variant as having abnormal intradermal histamine testing, which has only been associated so far with different forms of HSAN. We would like to highlight the importance of detailed and accurate description of clinical phenotyping of newly discovered genetic conditions, to have the best chance of matching a patient's clinical presentation with candidate genes found on genomic analysis.

## CONFLICT OF INTEREST

All authors declare that they have no conflict of interest or financial relationships that could be considered conflict of interest.
