# Supplementary material for: De novo pathogenic DNM1L variant in a patient diagnosed with atypical hereditary sensory and autonomic neuropathy
Source: Mol Genet Genomic Med. 2019 Sep 1;7(10):e00961. doi: 10.1002/mgg3.961 (PMC6785439; doi:10.1002/mgg3.961)
Supplement: Supplementary file 1 [file MGG3-7-e00961-s001.docx]

**Supplementary Appendix**

**TABLE OF CONTENTS**

Cover Page................................................................................................................................3

Supplementary Methods.....................................................................................................4-9

Case Presentation.............................................................................................4-6

Clinical Investigations....................................................................................6-8

Whole genome sequencing...............................................................................8-9

Supplementary Discussion................................................................................................9-10

Supplementary References.............................................................................................11-12

***Supplementary Appendix To:***

***De novo* pathogenic *DNM1L* variant in a patient diagnosed with atypical sensory autonomic neuropathy**

Tarailo-Graovac M^1,2^, Zahir FR^3,4^, Zivkovic I^5^, Moksa M^6^, Selby K^7^, Sinha S^6^, Nislow C^6^, Stockler-Ipsiroglu S^8^, Friedman JM^3^, van Karnebeek CDM^9,10^, Horvath GA^8^

^1^Departments of Biochemistry, Molecular Biology and Medical Genetics, Cumming School of Medicine, University of Calgary, Calgary, AB, Canada

^2^Alberta Children's Hospital Research Institute, University of Calgary, Calgary, AB, Canada

^3^Department of Medical Genetics, University of British Columbia, Vancouver, Canada

^4^College of Science and Engineering, Hamad Bin Khalifa University, Qatar

^5^University of British Columbia

^6^Faculty of Pharmaceutical Sciences, University of British Columbia, Vancouver, Canada

^7^Department of Pediatrics, Division of Pediatric Neurology, University of British Columbia

^8^Department of Pediatrics, Division of Biochemical Diseases, University of British Columbia

^9^Department of Pediatrics, Centre for Molecular Medicine and Therapeutics, BC Children’s Research Institute, University of British Columbia

^10^Departments of Pediatrics and Clinical Genetics, Academic Medical Centre, Amsterdam, The Netherlands

**Correspondence to:** Dr. Gabriella A. Horvath, Biochemical Diseases Division BC Children’s Hospital 4480 Oak Street Vancouver, BC, V6H 3V4, Canada. Tel: 604-875-2628; E-mail: [ghorvath@cw.bc.ca](mailto:ghorvath@cw.bc.ca) and Dr. Maja Tarailo-Graovac, Departments of Biochemistry, Molecular Biology and Medical Genetics, Alberta Children’s Hospital Research Institute (ACHRI), Cumming School of Medicine, University of Calgary, 3330 Hospital Drive NW, Calgary Alberta T2N 4N1, Canada. Tel: 403-210-9395; E-mail: [maja.tarailograovac@ucalgary.ca](mailto:maja.tarailograovac@ucalgary.ca).

**Supplementary methods**

**Case presentation**

This 10-year-old male was born as Twin B to Caucasian parents as the fifth child (Figure 1). The four older siblings, including his dizygotic twin, had no health concerns. The maternal serum triple screen and fetal ultrasound examinations in pregnancy were normal, and he was delivered at term via Caesarian section, weighing 2.7 kg. He remained in hospital for 10 days afterwards due to transient neonatal jaundice, hypoglycemia, poor feeding, and apnea. At discharge from hospital he was considered healthy.

Concerns regarding development were raised at the age of 4 months, when he was hypotonic, had swallowing difficulties and was behind in developmental milestones compared to his healthy twin. At 10 months of age, he was referred for assessment to Pediatric Neurology. From the age of 14 months he also showed self-injurious behavior, biting his fingers and toes without a visible pain response, and paroxysmal episodes of eye-rolling. He was started on an antiepileptic medication (Clobazam, Vigabatrin) due to an abnormal EEG and possible seizure activity.

At 24 months, the patient exhibited global developmental delay, decreased sensation and self-mutilating behavior; the possibility of cerebral palsy was suggested. He was sitting unsupported with crossed legs but had issues with truncal control. He did not transfer objects between hands although he could move his hands towards the midline. He could babble but was unable to say any words. He wore glasses to correct farsighted vision. He had no evidence of a known metabolic or neuromuscular disease, but his response to a growth hormone stimulation test was blunted, and low serum IGF1 level confirmed growth hormone (GH) deficiency. He was started on GH injections.

At age 3 years, the patient’s symptoms had worsened. He had severe lower limb hypertonia with exaggerated reflexes and received bilateral onabotulinumtoxinA injections with short-term relief. His self-injury worsened and his front teeth were removed at age 3 to protect his fingers and his tongue from being constantly chewed on.

At the age of 4 years, the patient developed myoclonic seizures and then generalized tonic-clonic seizures. His seizure frequency and duration increased over this year until he was admitted to Pediatric ICU in status epilepticus. He was intubated, ventilated and treated with continuous Midazolam infusion. His seizures were difficult to control, and he remained intubated for 3 weeks. During this hospital stay, which lasted 4 months, he was treated with several antiepileptic therapies (phenytoin, levetiracetam, topiramate, oxcarbazepine), but his seizures were poorly controlled, and he suffered hypoxic brain injury because of the prolonged seizure activity.

On discharge, he was transferred to a Pediatric rehabilitation facility, where he remained for another 2 months. He was wheelchair bound and needed complete care. He displayed signs of autonomic dysfunction, with episodes of severe tachycardia, heart rate in the 200’s and elevated temperature with flushing. He had sluggish reactive pupils, anisocoria, and was unable to fix or follow with his eyes. He had blue-colored, cold extremities. He also had severe apnea and was given oxygen by nasal prongs overnight to keep his oxygen saturation above 80%. He had increased secretions and aspirated frequently. He also developed severe dystonia and spasticity.

At age 5 years, the patient was admitted to hospital again with increasing frequency of seizures. In hospital lorazepam, levatiracetam, topiramate, and oxcarbazepine were added to his antiepileptic treatment regime.

At age 6 years he developed a spontaneous transverse fracture through the proximal tibial diametaphysis. Further fractures developed at age 7: angulated fracture at proximal tibial and fibular shafts; age 8 ½ : angulated, impacted distal right femoral fracture; and age 9: undisplaced Salter-Harris type 2 fracture of the distal metaphysis of the right tibia.

In the next 2 years his dystonia and spasticity progressed, and he developed contractures of his wrists and ankles. His dystonia improved slightly with trihexyphenidyl treatment, but this had to be stopped because of severe urinary retention. He also developed severe scoliosis and bilateral hip dislocation, but he was not considered a good candidate for surgical treatment.

At age 8 years he developed a pressure sore over the greater trochanter. He was noted to have equinovarus contractures in his feet, extension contractures of his knees, and adduction contractures in both hips. He had severe muscle wasting in his extremities. His tongue displayed severe damage due to self-mutilation, and the bottom four permanent incisors were removed. He had extensive corneal scarring because of dry eyes and inability to close the lids completely when asleep.

He had ongoing issues with temperature dysregulation. Examination showed that when he experienced temperature increases, he was unable to sweat.

His respiratory status and apnea worsened, and at age 10 years the patient died from respiratory insufficiency associated with an intercurrent illness. No autopsy was performed.

**Clinical Investigations**

***Brain MRI:*** The first MRI of his head at age 10 months showed lack of a posterior pituitary bright spot with no ectopic posterior pituitary, no visible anterior commissure, and possible optic nerve hypoplasia, suggestive of septo-optic dysplasia spectrum. There was also mild cerebellar atrophy. A repeat head MRI at age 2 ½ years demonstrated a pituitary gland with a normal bright spot and more prominent cerebellar atrophy. His MRS was normal. At the age of 4 years, while admitted to ICU, his MRI showed progressive cerebellar atrophy, and new signal abnormalities in the basal ganglia and possibly in the Rolandic cortex. His MRS showed an elevated lactate peak.

***EEG:*** An EEG at 10 months of age was abnormal with bilateral cerebral hemispheric slowing and multifocal spikes, suggesting predisposition to seizures. At age 2 years, EEG recorded in drowsiness and sleep was abnormal due to excessive slowing in multiple distributions and spike wave activity seen mainly in the anterior quadrants (R>L) and multifocal spikes. At the age of 5 years, the patient’s EEG showed severe diffuse encephalopathy, multifocal slowing, beta activity, and dysrhythmic background.

***Biochemical investigations:*** urine organic acids showed mild ketonuria and elevated lactate. Krabbe enzyme was normal. Plasma amino acids, VLCFA, serum transferrin isoelectric focusing and urine purines and pyrimidines were unremarkable. Sweat test was negative. IGF-1 was low at <1.4 (ref 4.1-21). Growth hormone arginine stimulation test was abnormal. CSF neurotransmitters showed low levels of dopamine metabolite and cerebral folate: HVA 218 nmol/L (ref 233-928), 5HIAA 83 nmol/L (ref 74-345), 5-methyltetrahydrofolate 34 nmol/L (40-150)**.** CSF GABA level was normal.

***Other tests:*** His spinal radiographs showed C-shaped thoracolumbar curvature facing to the right, progressing from Cobb angle 20^0^ at age 5 years to 85^0^ at age 10 years. Radiographs of the hips showed bilateral hip dislocations. Bone density performed at age 8 years with Hologic Discovery A bone densitometer showed fractures and low bone mass, with the lowest Z-score measurement of the total left hip of -4.2.

An intradermal histamine test (1:1000) at the age of 26 months showed normal weal but absent flare response, suggestive of an atypical HSAN. A sural nerve biopsy was inconclusive, while EMG and nerve conduction studies were suggestive of generalized axonal sensory neuropathy involving the upper and lower limbs equally.

An audiology exam showed limited behavioral responses at the age of 10 months.

***Targeted genetic investigations:*** there were no mutations found in *TWNK*, *ANT1*, *POLG1*, and urine *MELAS*, *MERRF* and *NARP* testing by long PCR was negative. Mitochondrial DNA sequencing was also negative.

**Whole genome sequencing**

***DNA Extraction from Saliva:*** Genomic DNA was extracted following DNA Genotek’s (DNAgenotek®) protocol for purification of whole sample. Final DNA concentration was quantified via absorbance using the NanoDrop Spectrophotometer (ND-1000).

***Whole genome library construction and sequencing:*** 2μg of purified gDNA was used for library generation using TruSeq DNA PCR-Free LT Library Preparation Kit (FC-121-3001, Illumina, USA) for an average insert size of 500 bp.  Agilent Bioanalyzer 2100 with High Sensitivity DNA Assay (Agilent, USA) and KAPA Library Quantification Kit – Illumina/Universal (KK4824, Kapa Biosystems, USA) were used to validate and quantify libraries. Pooled libraries were sequenced on an Illumina HiSeq 2500 (University of British Columbia) in rapid run or high output mode, generating 100 bp paired-end reads on three lanes of a flowcell for an average 30X coverage.

***WGS data analyses:*** The data were analyzed using our semi-automated bioinformatics pipeline [1]: (1) the Illumina sequencing reads were aligned to the human reference genome version hg19 using Bowtie 2 [2]; (2) the duplicates were marked and sorted using Picard; (3) variants were called using SAMtools and BCFtools after indel realignment using GATK; transcripts were annotated using SnpEff [3]; (4) functional variants were prioritized for rare variants by comparison against the public databases (dbSNP [4], NHLBI Exome Sequencing Project Exome Variant Server, and Exome Aggregation Consortium (ExAC) [5]); (5) both nuclear and mitochondrial genomes were analyzed and subsequently screened under a series of genetic models: mitochondrial, homozygous, hemizygous, compound heterozygous and *de novo* in search for candidate variants that could be reasonably assumed to contribute to the observed clinical phenotype.

***Sanger sequencing confirmation:*** Confirmation of the variants identified using WGS, as well as segregation with the disease was validated using Sanger sequencing at the CMMT/BCCHRI DNA Sequencing Core Facility. Sanger sequencing was performed on amplicons containing each nucleotide of interest using DNA from the available unaffected family members and the BigDye® Terminator v3.1 Cycle Sequencing chemistry (Life Technologies, USA). Subsequent capillary electrophoresis was performed by using a Prism 3130xl 16-capillary automated genetic analyzer (Applied Biosystems, USA).

**Supplementary DISCUSSION**

***HSAN*** is a group of disorders affecting the peripheral nervous system, predominantly the sensory and autonomic neurons. The most common symptoms are loss of sensation of pain and temperature. Patients often present with severe self-mutilating behavior, leading to ulcerations and soft tissue infections. Some patients have minor or more significant autonomic disturbances. Classification of the HSAN starts by dividing cases into two subgroups, based on their mode of inheritance. The autosomal dominant subgroup usually presents in adolescence or adulthood and includes the HSAN-I subtypes. The genes involved in this subgroup are: *SPTLC1, SPTLC2, ATL1, RAB7A* and *DNMT1*. The disease with autosomal recessive inheritance present much earlier, in infancy or childhood and includes HSAN-II (*WNK1, RETREG1, KIF1A*), HSAN-III (*IKBKAP*), HSAN-IV (*NTRK1*), HSAN-V (*NGFB*) and HSAN with spastic paraplegia (*CCT5*) [6].

After initial discovery of RETREG1 deficiency in HSAN type IIB (MIM 613115) patients [7], analysis of 75 unrelated individuals with clinical symptoms suggestive of HSAN revealed mutations in *RETREG1* in three additional families. All patients had ulcerations of hands and feet and osteomyelitis progressing to acro-osteolysis. Nerve conduction velocities showed axonal sensory neuropathy. Two more families were described later, and all patients presented with sensory and motor impairment of the lower limbs. Ulcerations, autonomic abnormality and spastic paraparesis were also present in one family [8] and loss of deep tendon reflexes and marked weakness in all muscle groups of the lower limbs in the other one. There was also scoliosis. Sural nerve biopsy showed a severely reduced density of large myelinated fibres [9].

The function of *RETREG1* is poorly understood. It is a component of the cis-Golgi matrix with a role in shaping and tethering the membrane stacks of its structure. The FAM134 family of proteins are endoplasmatic reticulum (ER) resident receptors that bind to autophagy modifiers and facilitate ER degradation by autophagy. The gene is predominantly expressed in sensory and autonomic ganglia. Down-regulation of FAM134B protein in human cells causes an expansion of the ER, while overexpression results in ER fragmentation and lysosomal degradation. Disruption of *Retreg1* in mice leads to apoptotic cell death and sensory nerve degeneration [10].

**Supplementary references**

1 Tarailo-Graovac M, Shyr C, Ross CJ, Horvath GA, Salvarinova R, Ye XC, Zhang L-H, Bhavsar AP, Lee JJY, Drögemöller BI, Abdelsayed M, Alfadhel M, Armstrong L, Baumgartner MR, Burda P, Connolly MB, Cameron J, Demos M, Dewan T, Dionne J, Evans AM, Friedman JM, Garber I, Lewis S, Ling J, Mandal R, Mattman A, McKinnon M, Michoulas A, Metzger D, Ogunbayo OA, Rakic B, Rozmus J, Ruben P, Sayson B, Santra S, Schultz KR, Selby K, Shekel P, Sirrs S, Skrypnyk C, Superti-Furga A, Turvey SE, Van Allen MI, Wishart D, Wu J, Wu J, Zafeiriou D, Kluijtmans L, Wevers RA, Eydoux P, Lehman AM, Vallance H, Stockler-Ipsiroglu S, Sinclair G, Wasserman WW, van Karnebeek CD. (2016). Exome Sequencing and the Management of Neurometabolic Disorders. *N Engl J Med*, **374**:2246–55.

2 Langmead B, Salzberg SL. (2012). Fast gapped-read alignment with Bowtie 2. *Nat Methods*, **9**:357–9.

3 Cingolani P, Platts A, Wang LL, Coon M, Nguyen T, Wang L, Land SJ, Lu X, Ruden DM. (2012). A program for annotating and predicting the effects of single nucleotide polymorphisms, SnpEff: SNPs in the genome of Drosophila melanogaster strain w1118; iso-2; iso-3. *Fly (Austin),* **6**:80–92.

4 Sherry ST, Ward MH, Kholodov M, Baker J, Phan L, Smigielski EM, Sirotkin K. (2001). dbSNP: the NCBI database of genetic variation. *Nucleic Acids Res*, **29**:308–11.

5 Lek M, Karczewski KJ, Minikel EV, Samocha KE, Banks E, Fennell T, O’Donnell-Luria AH, Ware JS, Hill AJ, Cummings BB, Tukiainen T, Birnbaum DP, Kosmicki JA, Duncan LE, Estrada K, Zhao F, Zou J, Pierce-Hoffman E, Berghout J, Cooper DN, Deflaux N, DePristo M, Do R, Flannick J, Fromer M, Gauthier L, Goldstein J, Gupta N, Howrigan D, Kiezun A, Kurki MI, Moonshine AL, Natarajan P, Orozco L, Peloso GM, Poplin R, Rivas MA, Ruano-Rubio V, Rose SA, Ruderfer DM, Shakir K, Stenson PD, Stevens C, Thomas BP, Tiao G, Tusie-Luna MT, Weisburd B, Won H-H, Yu D, Altshuler DM, Ardissino D, Boehnke M, Danesh J, Donnelly S, Elosua R, Florez JC, Gabriel SB, Getz G, Glatt SJ, Hultman CM, Kathiresan S, Laakso M, McCarroll S, McCarthy MI, McGovern D, McPherson R, Neale BM, Palotie A, Purcell SM, Saleheen D, Scharf JM, Sklar P, Sullivan PF, Tuomilehto J, Tsuang MT, Watkins HC, Wilson JG, Daly MJ, MacArthur DG. (2016). Exome Aggregation Consortium. Analysis of protein-coding genetic variation in 60,706 humans. *Nature*, **536**:285–91.

6 Rotthier A, Baets J, Timmerman V, Janssens K. (2012). Mechanisms of disease in hereditary sensory and autonomic neuropathies. *Nat Rev Neurol*, **8**:73–85.

7 Kurth I, Pamminger T, Hennings JC, Soehendra D, Huebner AK, Rotthier A, Baets J, Senderek J, Topaloglu H, Farrell SA, Nürnberg G, Nürnberg P, De Jonghe P, Gal A, Kaether C, Timmerman V, Hübner CA. (2009). Mutations in FAM134B, encoding a newly identified Golgi protein, cause severe sensory and autonomic neuropathy. *Nat Genet*, **41**:1179–81.

8 Ilgaz Aydinlar E, Rolfs A, Serteser M, Parman Y. (2014). Mutation in FAM134B causing hereditary sensory neuropathy with spasticity in a Turkish family. *Muscle Nerve*, **49**:774–5.

9 Murphy SM, Davidson GL, Brandner S, Houlden H, Reilly MM. (2012). Mutation in FAM134B causing severe hereditary sensory neuropathy. *J Neurol Neurosurg Psychiatry*. **83**:119–20.

10 Khaminets A, Heinrich T, Mari M, Grumati P, Huebner AK, Akutsu M, Liebmann L, Stolz A, Nietzsche S, Koch N, Mauthe M, Katona I, Qualmann B, Weis J, Reggiori F, Kurth I, Hübner CA, Dikic I. (2015). Regulation of endoplasmic reticulum turnover by selective autophagy. *Nature*, **522**:354–8.
